# Orange Seed Powder as a Novel Biosorbent for Congo Red Removal: Adsorption Mechanism, Isotherms, Kinetics, and Molecular Simulations

**DOI:** 10.3390/molecules31071152

**Published:** 2026-03-31

**Authors:** Baali Souad, Baali Kheira, Bourzami Riadh, Boudjema Lotfi, Laouet Nadjet, Saadi Sami, Boughellout Halima, Benatallah Leila

**Affiliations:** 1 Agri-Food Engineering (GENIAAL) Laboratory, Food Process Engineering, Biodiversity and Agro-Environment Team, Institute of Nutrition, Food and Agri-Food Technology (INATAA), Frères Mentouri Constantine 1 University (UFMC1), Constantine 25017, Algeria; baali.souad@umc.edu.dz (B.S.); saadi.sami@ymail.com (S.S.); halima.boughellout@umc.edu.dz (B.H.); leila.benatallah@umc.edu.dz (B.L.); 2 Department of Mechanical Engineering, 20 August 1955 University, P.O. Box 26, Skikda 21000, Algeria; baalikheira23@yahoo.fr; 3 Electromechanical Systems Laboratory (LSELM), Badji Mokhtar University, P.O. Box 12, Annaba 23000, Algeria; 4 Research Unit on Emergent Materials, University of Sétif 1, Setif 19000, Algeria; lotfi.boudjema@gmail.com; 5 Department of Chemistry, University College London, 20 Gordon St., London WC1E 6BT, UK; 6 Mathematical Physics and Subatomic Physics Laboratory (LPMPS), Frères Mentouri Constantine-1 University (UFMC1), Constantine 25017, Algeria; nadjet.laouet@umc.edu.dz

**Keywords:** orange seed powder, Congo red, adsorption, kinetics, isotherms, molecular simulation, waste valorization, biosorbent

## Abstract

The increasing discharge of synthetic dyes into industrial wastewater necessitates sustainable and low-cost treatment strategies. This study valorizes orange seed powder (OSP), an abundant agro-food residue, as a novel biosorbent for Congo red (CR) removal through a combined experimental and molecular simulation approach. Raw OSP was prepared solely by drying and grinding, without chemical activation, emphasizing its practical applicability and environmental sustainability. Physicochemical characterization using FTIR, SEM, and EDX confirmed adsorption-induced structural and compositional changes. Batch experiments evaluated the effects of initial dye concentration, adsorbent dosage, pH, temperature, and contact time. Equilibrium data were well fitted by the Langmuir and Freundlich isotherm models (*R*^2^ ≈ 0.99), with a maximum adsorption capacity of 258.39 mg g^−1^ at 25 °C and pH 4, and a removal efficiency exceeding 99.55%. The adsorption kinetics followed a pseudo-second-order model, while intraparticle diffusion contributed to the rate-controlling mechanism, as indicated by the Weber–Morris model. OSP demonstrated excellent regeneration performance over five adsorption–desorption cycles, retaining more than 96% of its initial CR removal efficiency when regenerated with methanol. Grand Canonical Monte Carlo (GCMC) simulations revealed that adsorption is primarily driven by electrostatic interactions, hydrogen bonding, and π–π stacking interactions, in good agreement with the experimental findings. Overall, raw OSP represents an efficient, regenerable, and sustainable biosorbent, highlighting the originality of integrating experimental investigations with GCMC simulations for wastewater treatment applications.

## 1. Introduction

Synthetic dyes are recognized as major environmental pollutants, and industrial effluents containing these compounds pose serious risks to public health and aquatic ecosystems [1]. Due to their extensive applications in industries such as textiles, cosmetics, paper, pharmaceuticals, and food processing [2], the release of wastewater loaded with dyes represents an important cause of environmental pollution, particularly aquatic system contamination [3]. These compounds are classified as persistent organic pollutants due to their chemical stability and resistance to biodegradation, and they present considerable ecotoxicological risks because of their mutagenic and carcinogenic potential, even at trace concentrations [4,5]. Consequently, the development of efficient and sustainable treatment strategies for hazardous dye-contaminated wastewater has become a major priority for environmental scientists and ecologists [3].

**Figure 1:**
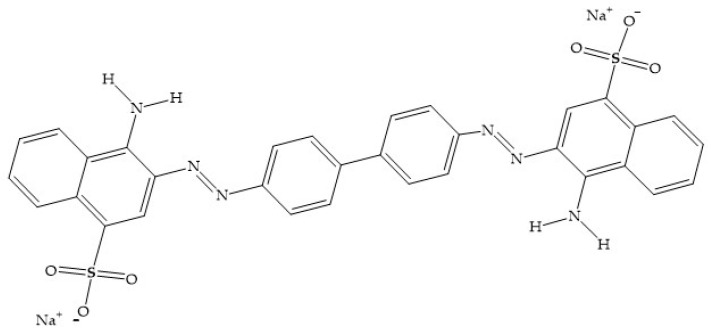
Developed chemical formula of CR.

Congo red (CR: 4-aminonaphthalene-1-sulphonic acid; Figure 1) is a synthetic anionic dye belonging to the polyazo group and is widely used in the textile, leather, pharmaceutical, cosmetic, and food industries [6,7]. Its molecular structure contains two azo chromophoric groups responsible for its intense coloration and ability to absorb ultraviolet and infrared radiation. Moreover, CR is highly water-soluble and exhibits strong persistence in natural environments [8,9]. Its toxicity and potential carcinogenicity raise serious concerns for human health and aquatic organisms [1]. Therefore, the efficient removal of CR from contaminated water has become a crucial challenge for environmental remediation efforts [10].

Several conventional methods have been employed for dye removal, including membrane filtration, reverse osmosis, chromatographic separation, and biological treatments, such as enzymatic degradation [11,12]. In addition, various physical and chemical techniques, including photocatalysis [13], ultrasound irradiation [14], ion exchange [15], coagulation–flocculation [16], advanced oxidation processes [17], and adsorption [18], have been investigated. Among these methods, adsorption is considered one of the most effective and versatile approaches due to its operational simplicity, cost-effectiveness, and minimal generation of secondary pollutants [19].

The adsorption performance strongly depends on the physicochemical properties of the adsorbent, such as particle size, surface chemistry, functional groups, and affinity toward dye molecules [1,20]. In this context, natural materials rich in lignin, cellulose, and humic substances have attracted considerable attention as sustainable adsorbents for azo dye removal [21]. Functional groups such as hydroxyl, carboxyl, carbonyl, and phenolic moieties play a key role in biosorption by enabling hydrogen bonding, electrostatic interactions, and π–π stacking interactions with pollutant molecules [1,21]. Consequently, the valorization of food and agricultural residues as bioadsorbents offers both environmental and economic advantages within a circular economy framework [22].

Numerous bio-based materials obtained from fungi, algae, and agricultural or agro-industrial residues have been explored as inexpensive adsorbent options, such as rice husk [23], jujuba seeds [24], *Eucalyptus globulus* sawdust [25], orange peel [26], *Aloe vera* leaves [7], sugarcane bagasse [27], pine bark [28], citrus residues [29], and pumpkin husk [30]. These materials are abundant, renewable, low-cost, and often rich in lignin and tannins, which are particularly favorable for dye adsorption [1,17,31]. Given their lignocellulosic composition and tannin content, orange seeds represent a promising but underexplored biomass for the adsorption of inorganic and organic pollutants from wastewater [7,18,30].

Indeed, encouraging results have been reported for the adsorption of anionic dyes, such as Reactive Orange 16, using orange seed-based materials [31]. Recent investigations have successfully demonstrated the potential of orange seed-based materials for dye removal. Flores Alarcón et al. [32] explored the use of defatted orange seed powder as a biosorbent for treating real textile wastewater, while Tahira et al. [33] synthesized iron oxide/activated charcoal nanocomposites from de-oiled orange seeds for Congo red adsorption. Although these studies confirm the viability of orange seeds, they rely on either lipid extraction with solvents [32] or energy-intensive pyrolysis and chemical impregnation [33] to enhance adsorptive properties. To date, no study has investigated the use of raw, unmodified orange seed powder as a low-cost adsorbent for Congo red removal.

In this context, the present study aims to valorize orange seeds (*Citrus sinensis*)—an abundant and underutilized agro-food residue from the citrus processing industry—by converting them into an efficient biosorbent through a simple and sustainable preparation strategy involving only washing, drying, and grinding, without any chemical activation, solvent extraction, or pyrolysis. This approach aligns with the principles of sustainable development and the circular economy, promoting the transformation of agricultural waste into value-added materials for environmental remediation. By directly valorizing this underutilized residue without energy-intensive pretreatments, we minimize the environmental footprint while maximizing resource efficiency.

Furthermore, this study provides a multifaceted analysis combining advanced experimental characterization techniques (FTIR and SEM–EDS), comprehensive kinetic and isotherm modeling, and Grand Canonical Monte Carlo (GCMC) molecular simulations. This integrated theoretical–experimental approach enables a deeper mechanistic understanding of the adsorption process on this specific lignocellulosic material. To the best of our knowledge, this is the first study to integrate experimental investigations with GCMC simulations for Congo Red adsorption onto raw orange seed powder.

Accordingly, the adsorption performance of orange seed powder for Congo red removal from aqueous solutions was evaluated using both experimental and theoretical approaches. Adsorption kinetics, equilibrium isotherms, and GCMC simulations were employed to elucidate the adsorption mechanism and provide a comprehensive understanding of the interactions governing the biosorption process.

## 2. Results and Discussion

### 2.1. FTIR 

**Figure 2:**
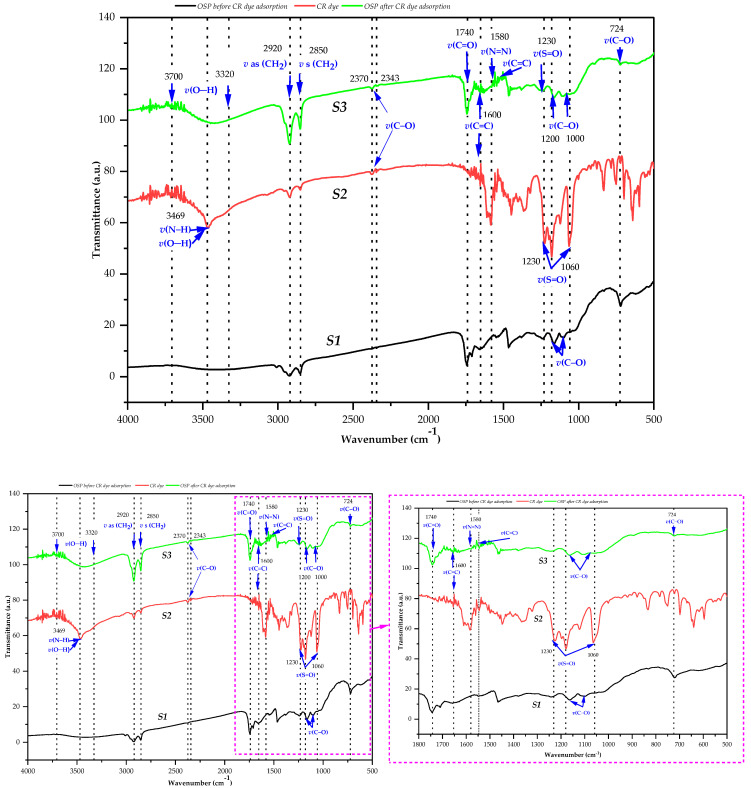
FTIR spectra of orange seed powder before adsorption (S1), Congo red dye (S2), and orange seed powder after Congo red adsorption (S3) under optimized conditions (initial CR concentration = 400 mg L^−1^, OSP dosage = 3 g L^−1^, pH = 4, contact time = 120 min, temperature = 25 °C, particle size = 125–500 µm). Pink dashed lines highlight the zoomed-in region between 500 and 1800 cm^−1^.

Fourier Transform Infrared Spectroscopy (FTIR) was employed to identify the surface functional groups of orange seed powder (OSP) before and after Congo red (CR) adsorption, and to characterize the pure dye (Figure 2). The FTIR spectra of raw OSP (S1), pure CR (S2), and CR-loaded OSP (S3) are presented in Figure 2, with a magnified region (1800–500 cm^−1^) highlighting key spectral changes. Comparative analysis of the spectra allows identification of band shifts, intensity variations, and the appearance or disappearance of characteristic adsorption bands, providing valuable insights into the interactions occurring at the biosorbent–adsorbate interface and helping to elucidate the potential binding sites involved in the adsorption process [34,35,36].

The FTIR spectrum of raw OSP (S1) exhibits typical features of lignocellulosic biomass materials [34,35]. The broad band at ~3320 cm^−1^ corresponds to O–H stretching vibrations of hydroxyl groups in cellulose, hemicellulose, and lignin. Adsorption bands at approximately 2920 and 2850 cm^−1^ correspond to asymmetric and symmetric stretching vibrations of aliphatic C–H groups. The band near 1740 cm^−1^ is assigned to C=O stretching vibrations of ester and carboxylic groups, mainly associated with hemicellulose and pectic substances. Additionally, the region between 1200 and 1000 cm^−1^, with a prominent band around 1020 cm^−1^, reflects C–O and C–O–C stretching vibrations of polysaccharides within the biomass matrix [34].

The FTIR spectrum of Congo red (S2) displays characteristic features of azo dyes [35,37]. Bands at ~1580 and 1505 cm^−1^ correspond to coupled stretching vibrations of aromatic C=C bonds and the azo linkage (–N=N–). Strong adsorption bands observed at ~1180, 1120, and 1035 cm^−1^ are attributed to S=O stretching vibrations of sulfonate (–SO_3_^−^) groups. Moreover, the band near 724 cm^−1^ corresponds to out-of-plane C–H bending vibrations of para-disubstituted aromatic rings, a diagnostic feature of Congo red [35].

After adsorption (S3), noticeable spectral modifications are observed, indicating the involvement of several functional groups in the adsorption process. The O–H stretching band broadens and shifts slightly to lower wavenumbers (~3305 cm^−1^), suggesting the formation of stronger hydrogen bonding interactions between hydroxyl groups of OSP and polar functional groups of CR [34,35]. A reduction in the intensity of the C=O band around 1740 cm^−1^ is also observed, indicating the involvement of carbonyl and carboxylic groups in dye binding through hydrogen bonding and electrostatic interactions [36,38].

Furthermore, characteristic adsorption bands of Congo red remain detectable in the S3 spectrum, confirming successful adsorption of the dye onto the OSP surface. Slight shifts in the sulfonate S=O stretching bands toward higher wavenumbers (~1188, 1128, and 1043 cm^−1^) reflects changes in the local chemical environment of sulfonate groups caused by to electrostatic interactions with OSP protonated surface sites under acidic conditions [35,38].

These observations are consistent with those reported by Tahira et al. [33], who also attributed Congo red adsorption onto orange seed-based nanocomposites to electrostatic interactions between surface functional groups and dye molecules. However, in contrast to their modified material (Fe_2_O_3_/AC), which exhibits characteristic Fe–O bands around 588 cm^−1^ [33], our raw OSP adsorbent requires no chemical functionalization to achieve remarkable adsorption efficiency. This comparison demonstrates the intrinsic potential of the native lignocellulosic matrix, as no metal–oxygen stretching vibrations are present in our spectra, confirming the absence of chemical modification.

Flores Alarcón et al. [32] employed defatted orange seed powder for the treatment of real textile wastewater and highlighted the importance of surface functional groups in the adsorption process. While their study focused on process optimization in complex effluent matrices, our FTIR results confirm and significantly extend these observations by precisely identifying, through quantitative shifts in the O–H, C=O, and S=O bands, the specific functional groups involved in the interaction with Congo red. This detailed mechanistic analysis at the molecular level, absent from previous studies [32,33], represents a significant advancement in understanding adsorption phenomena on this type of biosorbent.

A pronounced attenuation of the aromatic C–H out-of-plane vibration at ~724 cm^−1^ is observed after adsorption, reflecting the restricted vibrational motion of CR aromatic rings after binding to the biosorbent surface. This behavior is widely reported as spectroscopic evidence of strong surface interactions involving aromatic systems and supports the occurrence of π–π stacking interactions between the conjugated aromatic structure of Congo red and phenolic aromatic units of lignin present in OSP [37]. These π–π interactions are particularly relevant given the lignocellulosic composition of OSP, as previously documented in compositional studies of citrus seeds [39,40].

Overall, FTIR analysis indicates that Congo red adsorption onto orange seed powder occurs via a combined mechanism involving (i) hydrogen bonding between hydroxyl and carbonyl groups of OSP and polar functional groups of CR, (ii) electrostatic interactions between anionic sulfonate groups and protonated surface sites of the biosorbent, and (iii) π–π interactions between aromatic structures of CR and lignin moieties present in the OSP matrix. These interaction pathways are consistent with previously reported adsorption mechanisms for lignocellulosic biosorbents and explain the strong affinity of OSP toward Congo red removal [34,35,36,37,38].,in agreement with recent advances reported in the literature [41].

This mechanistic interpretation, supported by FTIR analysis and consistent with previous studies on orange seed-based adsorbents [31,32,33], highlights the intrinsic adsorption capability of raw OSP. The results demonstrate that unmodified OSP can act as an effective and sustainable biosorbent for dye removal, offering a promising low-cost alternative for wastewater treatment applications.

These findings are further supported by the GCMC simulations presented in the following section, which provide molecular-level insights into the adsorption interactions between CR molecules and the OSP surface.

### 2.2. SEM-EDS Analysis

**Figure 3:**
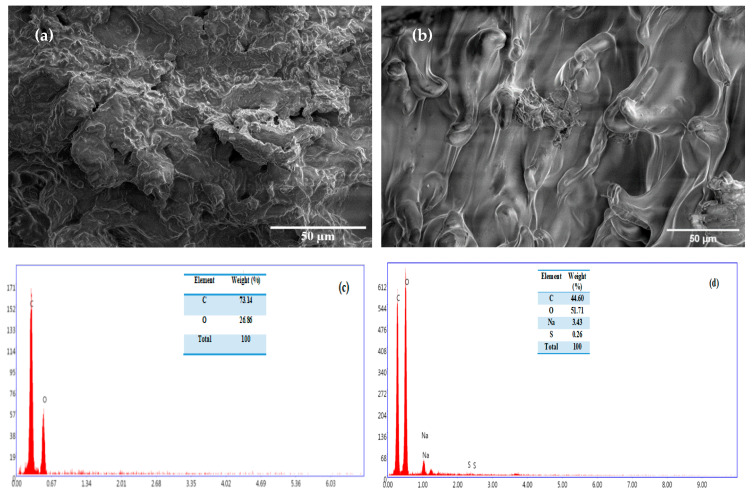
SEM images of OSP (**a**) before and (**b**) after CR-adsorption (magnification ×500), and their corresponding EDS spectra (**c**) and (**d**) respectively) under optimized conditions (initial Congo red (CR) concentration of 400 mg L^−1^, OSP dosage of 3 g L^−1^, pH 4, contact time of 120 min, temperature of 25 °C, and particle size range of 125–500 µm).

SEM micrographs of orange seed powder (OSP) before and after Congo red (CR) adsorption are presented in Figure 3a and Figure 3b, respectively. Prior to adsorption, OSP exhibits a rough and heterogeneous surface with a microporous morphology, which contributes to a relatively high surface area. This structure is typical of lignocellulosic biomaterials and is associated with the natural organization of cellulose, hemicellulose, and lignin within the plant matrix. The surface appears irregular and non-uniform, with numerous cavities and uneven features that are influenced by the intrinsic composition of the biomass and the preparation conditions of the adsorbent.

After CR adsorption (Figure 3b), noticeable changes in surface morphology are observed. The surface appears partially covered by a relatively smoother layer, indicating the deposition of dye molecules onto the adsorbent surface. In addition, some micropores become less visible or partially blocked, suggesting that CR molecules occupy available adsorption sites and diffuse into the porous structure of OSP. These observations provide morphological evidence supporting the adsorption process.

To verify that the observed surface layer corresponds to adsorbed CR molecules, energy-dispersive X-ray spectroscopy (EDS) analysis was performed (Figure 3c,d). The elemental composition of raw OSP confirms its organic nature, consisting mainly of carbon and oxygen. After adsorption, the appearance of sulfur (S, 0.26%) and sodium (Na, 3.43%) is clearly detected. These elements originate from the sulfonate groups (-SO_3_^−^Na^+^) present in the molecular structure of Congo red [42,43]. The detection of these elements therefore provides direct elemental evidence of CR adsorption onto the OSP surface, corroborating the functional group interactions identified by FTIR.

Furthermore, the decrease in carbon content (44.60%) accompanied by an increase in oxygen content (51.71%) can be attributed to the contribution of the adsorbed dye layer, which contains a significant proportion of oxygen-bearing functional groups. The EDS signal thus reflects the combined contribution of the OSP matrix and the CR molecules deposited on its surface. In this context, Na^+^ mainly acts as a counterion associated with the sulfonate groups of CR, indicating that its presence results from dye adsorption rather than from intrinsic components of the biosorbent.

Compared with previous studies, the present work provides direct elemental confirmation of dye attachment. For instance, Tahira et al. [33] characterized Fe_2_O_3_/activated carbon nanocomposites using physicochemical techniques but did not report EDS analysis to identify elemental markers of the adsorbed dye. In contrast, the detection of sulfur and sodium in the post-adsorption EDS spectrum in this study offers clear chemical confirmation of Congo red adsorption onto raw OSP.

Overall, the combined SEM–EDS characterization provides complementary morphological and elemental evidence supporting the successful adsorption of CR onto orange seed powder. These findings demonstrate that the intrinsic functional groups of the lignocellulosic matrix are sufficient to promote effective dye binding without the need for chemical modification, highlighting the potential of raw OSP as a low-cost and sustainable biosorbent for dye removal from aqueous solutions.

### 2.3. Effects of Operating Conditions on CR Adsorption

**Figure 4:**
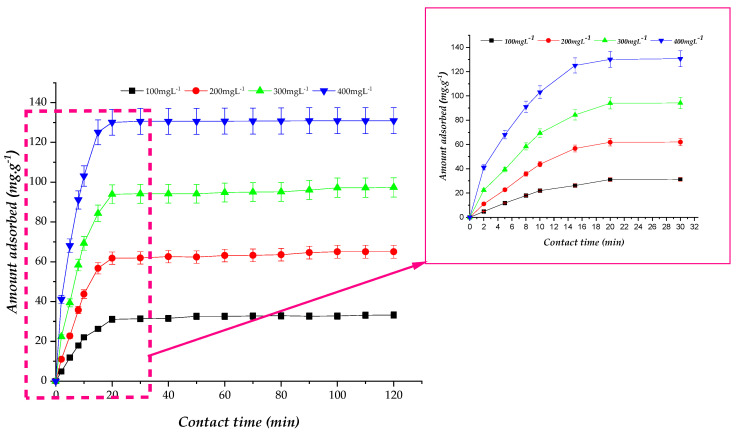
Effect of contact time on the adsorbed quantity of CR onto OSP (experimental conditions: OSP dose = 3 g L^−1^, pH = 6.95, temperature = 25 °C, stirring speed = 250 rpm, particle size = 125–500 µm). Results are expressed as mean ± standard deviation (n = 3). Dashed lines indicate the zoomed-in region between 0 and 32 min.

**Figure 5:**
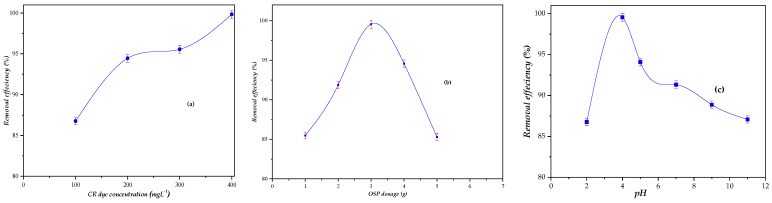
(**a**) Effect of initial CR concentration, (**b**) Effect of OSP concentration, (**c**) Effect of solution pH on the removal efficiency. (Non-variable used parameters: OSP dosage = 3 g L^−1^, CR-concentration = 400 mg L^−1^, pH = 6.95, agitation time 120 min, temperature 25 °C, stirring speed 250 rpm and particle size 125–500 µm.) Results are expressed as mean ± standard deviation (n = 3).

Adsorption performance was studied with respect to contact time, initial CR concentration, adsorbent concentration, and solution pH as illustrated in Figure 4 and Figure 5.

#### 2.3.1. Effect of Contact Time

Contact time is a key factor influencing the adsorption rate and removal efficiency of Congo red (CR) onto orange seed powder (OSP). It represents the time required to reach adsorption equilibrium and therefore governs the attainable adsorption capacity under given conditions. The effect of contact time was examined at various initial CR concentrations (100–400 mg.L^−1^), and the results are presented in Figure 4.

As shown in Figure 4, the adsorption of CR onto OSP exhibits a two-stage adsorption profile. During the initial 20 min, the adsorption rate is extremely rapid, with the adsorbed quantity reaching approximately 79–88% of the equilibrium capacity within the first 10 min, depending on the initial concentration. This initial phase is driven by the abundance of available active sites on the OSP surface, facilitating rapid uptake of dye molecules [44]. At this stage, the process is mainly governed by film diffusion, where CR molecules quickly migrate from the bulk solution to the adsorbent surface and occupy the easily accessible sites, driven by a high concentration gradient that favors external mass transfer.

Following this initial stage, the adsorption rate gradually decreases as the readily available sites become saturated. The subsequent slower phase is controlled by intraparticle diffusion, where CR molecules diffuse into the micropores of OSP until equilibrium is reached [45]. At equilibrium (approximately 90–120 min), the driving force for mass transfer diminishes, and further adsorption becomes negligible.

In summary, the adsorption kinetics indicate that CR uptake onto OSP initially occurs through rapid surface adsorption followed by a slower diffusion-controlled process within the adsorbent pores. This behavior is consistent with adsorption mechanisms commonly reported for lignocellulosic biosorbents [44,45].

#### 2.3.2. Effect of Initial CR Concentration

The initial concentration of Congo red (CR) is a key parameter affecting adsorption performance, as it governs the mass transfer driving force between the solution and the adsorbent surface. Batch experiments were performed at initial CR concentrations of 100–400 mg L^−1^, and the results are shown in Figure 5a.

The removal efficiency increased from 86.76% to 99.83% as the initial CR concentration increased from 100 to 400 mg L^−1^. This behavior can be explained mechanistically: higher initial concentrations provide a greater concentration gradient, enhancing the diffusion of CR molecules toward the OSP surface and promoting interaction with active sites [45,46].

The adsorbent dosage used in this study was sufficient to accommodate the increased dye loading, resulting in high removal efficiencies across the investigated concentration range.

No precipitation, critical concentration, or “supersaturation” phenomena were observed. The adsorption capacity of OSP increased with the initial CR concentration because more dye molecules were available to occupy the active sites, until the surface sites approached saturation. This interpretation is consistent with established adsorption theory, where mass transfer and site availability control uptake rather than solubility or aggregation effects [42,46].

In summary, the effect of initial CR concentration demonstrates that higher concentrations enhance the adsorption driving force, leading to increased removal until the adsorbent reaches its maximum adsorption capacity.

#### 2.3.3. Adsorbent Dosage Effect

The dosage of orange seed powder (OSP) is a critical factor influencing the adsorption of Congo red (CR). Batch experiments were conducted under optimized conditions to evaluate the effect of varying OSP doses on removal efficiency, as shown in Figure 5b.

The results indicate that the removal efficiency of CR increases with increasing OSP dosage, achieving a maximum removal efficiency at a dosage of 3 g L^−1^. This behavior can be attributed to the greater availability of adsorption sites and surface area at higher adsorbent doses, which facilitates the uptake of more CR molecules from the solution [43,47]. Beyond the optimal adsorbent dosage, further increases do not significantly improve removal efficiency because the accessible adsorption sites in the solution become saturated, or slight aggregation of OSP particles may reduce the effective surface area available for adsorption. This observation is consistent with classical adsorption theory, where the absorbent dosage governs the number of active sites available for interaction with adsorbate molecules [43,48].

In conclusion, 3 g L^−1^ was identified as the optimal OSP dosage, yielding the highest removal efficiency under the experimental conditions. Additionally, the initial particle size of OSP can influence adsorption efficiency, as smaller particles provide a larger surface area and greater accessibility of adsorption sites, thereby enhancing CR uptake.

#### 2.3.4. Effect of Initial Solution pH

The initial pH of the Congo red (CR) solution is a critical parameter influencing adsorption, as it affects both the surface charge and functional group ionization of OSP as well as the ionization state of the dye [49,50,51,52]. The effect of pH was investigated within a pH range of 2–11 at 25 °C for 120 min, and the results are shown in Figure 5c.

As shown, the removal efficiency of CR was 86.76% at pH 2, increased to a maximum of 99.55% at pH 4, and decreased progressively at higher pH values. This behavior can be mechanistically explained by the electrostatic interactions between CR molecules and OSP functional groups. At pH values below the point of zero charge of OSP (pH_PZC ≈ 5), the surface carries a net positive charge due to protonation of –OH and –COOH groups. The negatively charged sulfonate groups (–SO_3_^−^) of CR are attracted electrostatically to the positively charged OSP surface, thereby enhancing adsorption efficiency. Hydrogen bonding between CR functional groups and oxygen-containing groups on OSP may also contribute to adsorption at acidic pH [53,54].

At pH values above 5, deprotonation of –COOH and –OH groups results in a negatively charged OSP surface. Electrostatic repulsion between the negatively charged OSP and anionic CR reduces adsorption and accounts for the observed decrease in removal efficiency. Despite this, OSP still achieves significant CR removal even at pH 9 (88.86%) and pH 11 (87.07%), demonstrating its robustness across a wide pH range.

These findings are consistent with previous reports on biosorbents such as coir pith carbon [43], rice husk ash [54], and neem leaf powder [55], where maximal adsorption is generally achieved under mildly acidic conditions, with diminished efficiency at higher pH due to electrostatic repulsion. Overall, the results confirm that OSP is an effective and versatile adsorbent for CR across diverse pH conditions.

### 2.4. Adsorption Isotherms

Adsorption isotherms play a crucial role in understanding equilibrium adsorption behavior and the interactions between adsorbate and adsorbent. In this study, the Langmuir and Freundlich isotherm models were employed to describe the adsorption of Congo red (CR) onto orange seed powder (OSP). These models enable estimation of adsorption capacity and provide insights into the affinity between CR molecules and the OSP surface.

**Figure 6:**
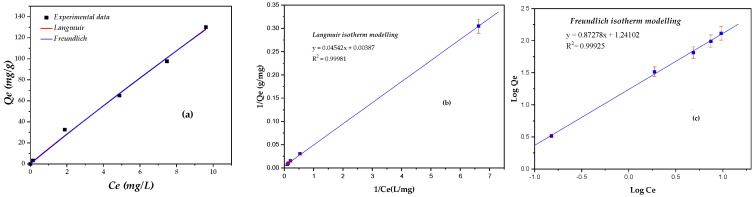
Isotherm modelling of CR adsorption on OSP (**a**) nonlinear fitted curve with isotherm models of Langmuir and Freundlich, (**b**) Langmuir, (**c**) Freundlich (OSP/CR: 3 g/L, pH = 4, temperature 25 °C, stirring speed 250 rpm and particle size 125–500 µm).

The equilibrium adsorption data were analyzed using both models, and the corresponding parameters are summarized in Table 1. The Langmuir and Freundlich isotherms are widely applied in biosorbent systems to reveal surface properties and adsorption mechanisms, including surface homogeneity and heterogeneity [56,57,58,59]. Figure 6a presents the experimental equilibrium data along with the fitted Langmuir and Freundlich isotherm curves for CR adsorption onto OSP.

Both models exhibited very high correlation coefficients (*R*^2^ > 0.999), indicating a strong agreement with the experimental equilibrium data. However, as recommended, goodness of fit was assessed using more than just linearized *R*^2^ values. The chi-square (χ^2^) statistical test was also employed to assess the deviation between experimental (*Q_e,exp_*) and calculated (*Q_e,cal_*) adsorption capacities, providing a more rigorous comparison of model performance.

#### 2.4.1. Langmuir Isotherm

The Langmuir isotherm model is designed to study monolayer adsorption processes. It assumes that adsorption occurs on a uniform, homogenous surface with equivalent sites on the adsorbent [56]. The Formulas (1) and (2) represent, respectively, the nonlinear and linearized forms of the Langmuir isotherm Equation:

(1) $$Q_{e}=\frac{\left(Q_{m}\times b\times C_{e}\right)}{1+\left(b\times C_{e}\right)}$$

(2) $$\frac{1}{Q_{e}}=\frac{1}{Q_{m}}+\left(\frac{1}{Q_{m}\times b}\right)\frac{1}{C_{e}}$$

where $Q_{e}$ is the adsorption capacity of CR at the equilibrium (mg.g^−1^), $C_{e}$ is the equilibrium concentration of CR (mg.L^−1^), $Q_{m}$ represents the maximum adsorption capacity corresponding to complete monolayer coverage (mg.g^−1^), and b is the Langmuir equilibrium constant (L.mg^−1^), which is proportional to the apparent sorption energy. The Langmuir isotherm parameters $Q_{m}$ and b were determined from the slope and intercept of the plot $\frac{1}{Q_{e}}\;vs.\frac{1}{C_{e}}$. The fit of experimental data to the Langmuir model is shown in Figure 6b, and the corresponding Langmuir constants are summarized in Table 1.

The Langmuir isotherm is based on the concept of single-layer adsorption occurring on a surface that contains a limited set of uniform, energetically equivalent sites, assuming no interactions between adsorbed species [56]. The Langmuir parameters obtained in this study indicate a high apparent monolayer adsorption capacity of OSP toward CR ($Q_{m}$ = 258.39 mg g^−1^), highlighting the strong affinity between the dye molecules and the biosorbent surface.

An essential characteristic of the Langmuir model is the dimensionless separation factor ($R_{L}$), calculated using the following Equation (3):

(3) $$R_{L}=\frac{1}{1+bC_{i}}$$

where $C_{i}$ is the initial CR concentration and b is the Langmuir constant. The value of $R_{L}$ indicates the possibility of adsorption on a single layer and the adsorption nature to be either unfavourable ($R_{L}>1$), linear ($R_{L}=1$), favorable (${0<R}_{L}<1$) or irreversible ($R_{L}=0$).

The dimensionless separation factor ${(R}_{L})$, calculated for the investigated initial CR concentrations, ranged from 0.0285 to 0.1050 (Table 1), confirming that the adsorption process is favorable over the entire concentration range. Similar favorable Langmuir behavior has been reported for CR adsorption onto other lignocellulosic biosorbents, such as cabbage waste powder [46], coir pith [43], and Phoenix dactylifera seeds [45].

Despite the excellent correlation coefficient *R*^2^ = 0.99981), noticeable discrepancies between *Q_e,exp_* and *Q_e,cal_*, as well as relatively higher χ^2^ values, were observed at higher concentrations. These deviations can be attributed to the idealized assumptions of the Langmuir model, which are not fully consistent with the chemically and structurally heterogeneous nature of raw orange seed powder. As reported in previous studies [34,60,61] lignocellulosic biosorbents possess diverse functional groups and non-uniform adsorption energies, which may lead to departures from ideal monolayer behavior. Therefore, in this study, the Langmuir model is best interpreted as providing an apparent monolayer capacity that is useful for comparison purposes rather than as evidence of a perfectly homogeneous adsorption surface.

#### 2.4.2. Freundlich Isotherm

For non-ideal adsorption on heterogeneous surfaces and possible multilayer adsorption, the Freundlich isotherm model is commonly applied [59]. The Freundlich model is an empirical equation that assumes adsorption occurs on sites with varying adsorption energies. The nonlinear and linear forms of the Freundlich equation are given by Equations (4) and (5), respectively:

(4) Qe=KfCe1n

(5) $${LogQ}_{e}=LogK_{f}+\left(\frac{1}{n}\right)LogC_{e}$$

where $K_{f}$ (mg g^−1^)(L.mg^−1^)^1/*n*^ is the Freundlich constant related to adsorption capacity, and n is the adsorption intensity parameter. Values of n>1 reflect favorable adsorption. The parameters $K_{f}$ and n were determined from the intercept ($LogK_{f}$) and slope $\left(\frac{1}{n}\right)$ of the linear plot of ${LogQ}_{e}$ vs. $LogC_{e}$.

The plot of ${LogQ}_{e}$ vs. $LogC_{e}$ for CR adsorption onto OSP is shown in Figure 6c, and the corresponding Freundlich constants are summarized in Table 1.

For this system, the Freundlich model yielded an adsorption intensity of n=1.1457(n>1), indicating favorable adsorption and supporting the heterogeneous nature of the OSP surface. This suggests that CR molecules interact with OSP through adsorption sites with different energies, which is consistent with the lignocellulosic composition of the biosorbent [59].

The heterogeneity factor (1/*n* = 0.8727) reflects a non-uniform distribution of adsorption energies, further confirming the heterogeneous surface of OSP. Additionally, the Freundlich model yielded lower χ^2^ values and calculated adsorption capacities closer to experimental data compared to the Langmuir model (Table 1), suggesting a more realistic description of CR adsorption on OSP. Similar behavior has been reported for other heterogeneous biosorbents [35,37,46].

Overall, the isotherm analysis indicates that Congo red (CR) adsorption onto orange seed powder (OSP) occurs on a heterogeneous surface with energetically non-equivalent sites. The process is predominantly governed by physical interactions, including electrostatic attraction and hydrogen bonding, as corroborated by FTIR analysis. While both Langmuir and Freundlich models describe the equilibrium data adequately, the Freundlich model better captures surface heterogeneity and adsorption capacities closer to experimental values. The Langmuir model remains useful for estimating the theoretical maximum adsorption capacity and for comparing OSP performance with other reported biosorbents [24,26,42,43,46,53].

The adsorption capacity of OSP ($Q_{m}$ = 258.39 mg g^−1^) is higher than that of most other low-cost agricultural and industrial biosorbents (Table 2). For example, Ackee apple seeds [53] and Phoenix dactylifera seeds [45] achieved $Q_{m}$ values of 161.89 mg g^−1^ and 61.72 mg g^−1^, respectively, while modified orange peel powder reached 107 mg g^−1^ [26]. This superior performance can be attributed to the heterogeneous lignocellulosic structure of OSP, providing a diverse distribution of adsorption sites and functional groups favorable for interactions with CR molecules.

The Langmuir separation factor ($R_{L}$) for OSP (0.0285–0.105) indicates strongly favorable adsorption over a wide concentration range, while the Freundlich exponent (*n* = 1.1457) confirms both adsorption intensity and surface heterogeneity, consistent with the biosorbent’s lignocellulosic composition. Compared to more uniform adsorbents such as Cabbage Waste Powder ($R_{L}$= 0.0086–0.0801, *n* = 1.568) [46] and coir pith (*n* = 3.39–4.78) [43], OSP demonstrates heterogeneous adsorption sites and potential multilayer adsorption behavior, supported by its fit to both Langmuir and Freundlich models.

The exceptional performance of OSP becomes even more evident when compared directly with modified orange seed-based adsorbents recently reported in the literature. Tahira et al. [33] synthesized iron oxide/activated charcoal nanocomposites (Fe_2_O_3_/AC) from de-oiled orange seeds for Congo red adsorption. Their material, despite requiring energy-intensive pyrolysis at 450 °C followed by chemical impregnation, exhibited a maximum adsorption capacity of only 9.21 mg g^−1^ [33]. In contrast, the raw, unmodified OSP used in this study achieved a $Q_{m}$ value of 258.39 mg g^−1^—approximately 28 times higher—while requiring no chemical treatment, de-oiling, or thermal activation. 

A recent review by Manzoor et al. [41] highlighted an important gap in the field, noting that although numerous plant-based biosorbents have been investigated for Congo red removal, relatively few studies have examined unmodified agro-industrial residues with detailed mechanistic analysis. The present work contributes to filling this gap by demonstrating that raw orange seed powder not only matches but, in many cases, surpasses the performance of modified adsorbents. These results challenge the prevailing assumption that chemical or thermal activation is necessary to achieve high dye adsorption efficiency.

This remarkable difference highlights the exceptional intrinsic affinity of the native lignocellulosic matrix for Congo red. The superior performance of raw OSP can be attributed to the preservation of its native functional groups—particularly hydroxyl (–OH), carboxyl (–COOH), and phenolic moieties—which serve as effective binding sites for CR molecules through hydrogen bonding and electrostatic interactions [24,26]. In addition, the retention of the original porous architecture ensures optimal accessibility to these active sites, whereas thermal or chemical modification may collapse pore structures or consume functional groups, thereby reducing adsorption capacity [41]. These findings suggest that the harsh modification conditions employed in previous studies may not only be unnecessary but could also diminish the natural adsorption potential of the biomass.

Overall, Table 2 demonstrates that OSP exhibits superior adsorption capacity and favorable adsorption characteristics over a broad range of initial concentrations, highlighting its potential as an efficient, low-cost, and sustainable adsorbent for CR removal. The data emphasize the importance of surface heterogeneity, functional group availability, and biosorbent structure in maximizing adsorption efficiency, aligning with trends observed in other lignocellulosic biosorbents [24,26,43,45,46,53].

### 2.5. Adsorption Kinetics

**Figure 7:**
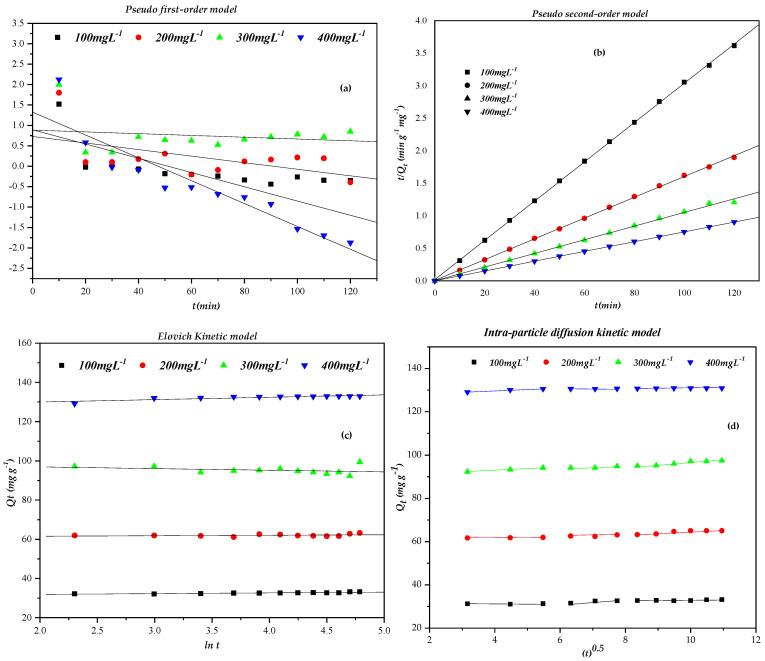
Fitting of experimental data by the models: (**a**) Pseudo-first-order, (**b**) Pseudo-second-order, (**c**) Elovich kinetic, (**d**) Intra-particle diffusion (OSP/CR = 3 g/L, pH = 4, temperature = 25 °C, stirring speed = 250 rpm and particle size = 125–500 µm).

The removal mechanism of CR from aqueous media, along with the reaction pathways and possible rate-controlling steps, was investigated using several kinetic models, including the Lagergren pseudo-first-order, pseudo-second-order, Elovich, and Weber–Morris intraparticle diffusion models [31,64,65,66,67,68,69,70,71,72]. Table 3 lists the kinetic parameters for each model, calculated from the intercepts and slopes of the corresponding linear plots (Figure 7), with *R*^2^ indicating the goodness of fit. This analysis provides valuable insights into the underlying mechanisms driving the adsorption process and highlights the rate-controlling steps.

Comparison of the kinetic data presented in Table 3 and the graphical representations in Figure 7 leads to the following conclusions:

The linear plots of $Log\left(Q_{e}-Q_{t}\right)$ vs. t (Figure 7a) yielded straight lines that do not intersect the origin, with correlation coefficients ranging from 0.826 to 0.891 (Table 3). Moreover, the calculated equilibrium adsorption capacities $Q_{e,cal}$ were drastically lower than the experimental values $Q_{e,exp}$ (e.g., 2.27 vs. 32.71 mg g^−1^ at 100 mg L^−1^), and the chi-square (χ^2^) values were extremely high (e.g., 408.59 at 100 mg L^−1^). Additionally, the rate constant $K_{1}$ exhibited negative values, which are physically meaningless. These results clearly demonstrate that the pseudo-first-order model is inadequate for describing the adsorption of Congo red (CR) onto orange seed powder (OSP).

As shown in Figure 7b, the plots of (tQt) versus t are linear over the whole concentration range, with *R*^2^ values very close to unity (0.9997–1.0000). The calculated $Q_{e,cal}$ values are in excellent agreement with the experimental ones, and the χ^2^ values are negligibly small (Table 3). This demonstrates that the pseudo-second-order model accurately describes the kinetics of CR adsorption onto OSP. This model assumes that the rate-limiting step may involve chemisorption through sharing or exchange of electrons between adsorbate and adsorbent, although physical interactions (hydrogen bonding, van der Waals) can also contribute to the overall uptake [31,46,73,74]. The rate constant *k_2_* generally decreased with increasing initial CR concentration (from 0.0264 to 0.0098 g mg^−1^ min^−1^), except at 400 mg L^−1^ where a higher value (0.0711 g mg^−1^ min^−1^) was observed; this anomaly might be related to a change in the adsorption mechanism at high dye loadings (e.g., multilayer formation or pore filling). Studies by Rojas [75] indicate that the number of active sites on the sorbent is proportional to the adsorption capacity, and the CR-adsorption reaction with the adsorbent controls the mechanism rather than mass transfer. Likewise, numerous earlier investigations have reported that the pseudo-second-order model provides an accurate description of CR adsorption across a variety of adsorbent materials [7,31,46,53,58,76].

Figure 7c presents the Elovich model fitting of the adsorption kinetics. The parameters α and β were obtained from the linear plot of $Q_{t}$ vs. lnt, yielding correlation coefficients in the range 0.8840 ≤ *R*^2^ ≤ 0.9283 (Table 3). These R^2^ values are lower than those obtained for the pseudo-second-order model, indicating that the Elovich model provides a less satisfactory fit. According to the Elovich equation, α represents the initial adsorption rate, and β is related to surface coverage and activation energy for chemisorption. The α values increased with increasing initial CR concentration (up to 10^88^ mg g^−1^ min^−1^ at 400 mg L^−1^), which may reflect the enhanced driving force for mass transfer at higher concentrations rather than solely an increase in the number of active sites [77]. The β values decreased with increasing CR concentration up to 300 mg L^−1^ and then varied at 400 mg L^−1^, suggesting changes in surface heterogeneity and site availability [46,78]. The reciprocal values (1/β) at 100, 200, 300, and 400 mg L^−1^ were 0.93, 1.46, 1.98, and 0.62, respectively; however, these values should not be directly interpreted as the absolute number of adsorption sites, but rather as an empirical indication of surface heterogeneity. Furthermore, the non-zero intercepts of the Elovich plots indicate that adsorption is not governed solely by chemisorption. Instead, the overall adsorption mechanism likely involves multiple steps, including external mass transfer and intraparticle diffusion. These observations, together with the Weber–Morris analysis, suggest that intraparticle mass transport contributes to the adsorption rate but is not the only rate-controlling mechanism.

The Weber–Morris intraparticle diffusion model was applied to further elucidate the adsorption mechanism of Congo red onto orange seed powder (Figure 7d). According to Table 3 and Figure 7d, the plots of $Q_{t}$ vs. *t*^0.5^ exhibit multi-linear behavior with three distinct segments, indicating that the adsorption process involves multiple steps [79,80,81].

The first segment ($t^{0.5}<6\;{min}^{0.5}$) corresponds to instantaneous external surface adsorption or film diffusion, where CR molecules rapidly occupy the readily available active sites on the outer surface of OSP. This stage is controlled by mass transfer from the bulk solution to the adsorbent surface.

The second segment $\left(6\;{min}^{0.5}<t^{0.5}<8\;{min}^{0.5}\right)$ is attributed to gradual intraparticle diffusion, where CR molecules diffuse into the interior pores of OSP. The intraparticle diffusion rate constant ($k_{d}$) increased from 0.289 to 0.629 mg g^−1^ min^−0.5^ as the initial CR concentration increased from 100 to 300 mg L^−1^, reflecting the enhanced mass transfer driving force at higher concentrations. However, a decrease in $k_{d}$ was observed at 400 mg L^−1^ (0.175 mg g^−1^ min^−0.5^), which may be associated with partial pore saturation or increased diffusion resistance within the adsorbent matrix.

The third segment ($t^{0.5}>8\;{min}^{0.5})$ represents the final equilibrium stage, where adsorption becomes negligible due to saturation of available active sites and low residual dye concentration.

The intercept values ($C_{d}$), related to the boundary layer thickness, increased progressively from 30.06 to 129.22 mg g^−1^ with increasing initial concentration (Table 3). Larger $C_{d}$ values indicate a thicker boundary layer, implying greater resistance to external transfer [75,79]. The correlation coefficients ranged from 0.708 to 0.928, and importantly, none of the linear segments passes through the origin, confirming that intraparticle diffusion is not the sole rate-limiting step and that film diffusion contributes significantly to the adsorption process.

Overall, the adsorption kinetics are best described by the pseudo-second-order model, although the contributions of film diffusion and intraparticle diffusion remain significant throughout the process [80,81]. A comparable multi-stage adsorption mechanism was reported by Suteu et al. for Orange-16 dye removal using OSP [31], and similar findings have been documented in other studies [46,48,82,83,84].

### 2.6. Proposed Adsorption Mechanism

In this section, we examine the mechanisms governing the adsorption of Congo red (CR) and water onto orange seed powder (OSP). Specifically, we analysed the adsorption behavior at both low initial concentrations and at saturation by employing the Grand Canonical Monte Carlo (GCMC) approach to elucidate the underlying microscopic processes. The integration of GCMC modeling with adsorption isotherms provided detailed insights into site-specific interactions, and pore occupation, offering a comprehensive understanding of CR adsorption on OSP and valuable guidance for optimizing its use as a sustainable adsorbent.

The adsorption mechanism of Congo red (CR) onto orange seed powder (OSP) is revealed to be a complex, multi-step process, which has been elucidated through both experimental observations and theoretical simulations. A comprehensive suite of analytical techniques—including Fourier Transform Infrared Spectroscopy (FTIR), Scanning Electron Microscopy coupled with energy dispersive X-ray spectroscopy (SEM-EDS), adsorption isotherm modeling, and kinetic studies—demonstrate that several types of molecular interactions act in synergy during CR adsorption. In addition to the experimental findings, Grand Canonical Monte Carlo (GCMC) simulations offer detailed insight into the microscopic dynamics of the system, allowing a deeper understanding of CR interactions with the adsorbent across low and high concentration ranges [60,85].

At the molecular level, FTIR analyses indicate significant shifts in the adsorption bands after CR adsorption. Notably, the reduction in intensity of the broad O–H stretching bands (3700–3100 cm^−1^) suggests that hydroxyl groups on OSP are involved in hydrogen bonding with CR. Furthermore, the emergence of an NH_2_ stretching band at 3469 cm^−1^, which was absent in the pristine OSP spectrum provides clear evidence that CR’s amino groups are interacting with active surface sites. In addition, alterations in the spectral regions corresponding to carbonyl (–C=O), aromatic (–C=C–), azo (–N=N–), and sulfonate (–S=O) functionalities underscore the formation of a complex adsorption network dominated by physical interactions and strong electrostatic attractions. SEM images reveal that the original rough, microporous structure of OSP, which offers a high specific surface area, becomes noticeably smoother after CR uptake, suggesting that a CR layer has formed, filling the micropores. EDS further confirms these findings by detecting sulfur and sodium on the CR-treated surface, elements inherent to the dye [86].

**Figure 8:**
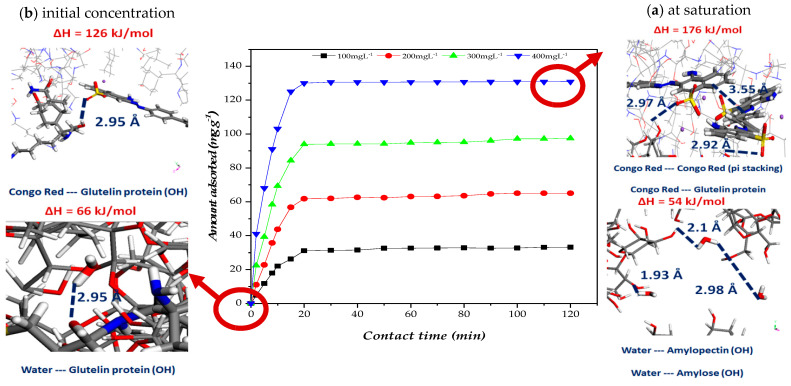
Adsorption isotherm curves from experimental data. (**a**) Primary interactions at saturation: This panel illustrates the key interactions between CR and water molecules with the orange seed structure model at saturation conditions. The system consists of 2 CR molecules and 10 water molecules per unit cell, corresponding to an approximate adsorption capacity of 130 mg/g. Each molecule’s interaction with the amorphous matrix is analysed individually to highlight specific adsorption sites and binding mechanisms. (**b**) Primary interactions at initial concentration: This panel depicts the fundamental interactions under initial concentration conditions, where 1 CR molecule and 1 water molecule are individually assessed in relation to the orange seed structure model. This setup allows for the examination of isolated adsorption dynamics, providing insight into the competitive and cooperative effects observed during the early stages of adsorption. Atom colors: C = gray, O = red, H = white, N = blue. Dashed lines indicate hydrogen bonds between water and –OH groups of glutelin or amylose.

GCMC simulations offer valuable theoretical insights into the adsorption process under varying conditions (Figure 8). Under low-concentration conditions (Figure 8a), where approximately one CR molecule is present per unit cell along with one water molecule, the simulations show that localized interactions predominate. Here, high-energy adsorption sites on OSP are rapidly occupied by CR primarily through electrostatic attractions and hydrogen bonding, ensuring that CR preferentially binds even in the presence of competing water molecules. At saturation (Figure 8a), with around two CR molecules and ten water molecules per unit cell—corresponding to an approximate adsorption capacity of 130 mg g^−1^—the adsorption mechanism becomes governed by an extensive network of interactions. In these conditions, the protonation of OSP’s functional groups (such as –OH and –COOH) under acidic conditions (pH 4) creates positively charged sites that attract the negatively charged sulfonate groups of CR. Additionally, multiple hydrogen bonds and π–π stacking interactions between CR’s aromatic rings and the aromatic components of the lignin-rich OSP further stabilize the adsorbed layer, while pore-filling effects maximize the available surface area for adsorption [87].

By integrating both experimental observations and molecular simulation results, the overall adsorption mechanism of Congo Red (CR) onto orange seed powder (OSP) can be described in three distinct stages. At low CR concentrations, rapid adsorption occurs through the occupation of high-energy active sites, primarily driven by electrostatic attractions and hydrogen bonding. This is facilitated by mass transfer mechanisms such as film diffusion and intraparticle diffusion, which transport CR molecules to the biosorbent surface. As adsorption progresses toward equilibrium, a monolayer of CR forms on the OSP surface. At higher concentrations, additional adsorption is observed either via multilayer formation or pore filling, further stabilized by π–π stacking interactions between the aromatic rings of CR and the lignin-rich domains of OSP. These phenomena are consistent with the adsorption capacities described by both the Langmuir and Freundlich models, highlighting the system’s combination of monolayer and heterogeneous adsorption behavior.

From a thermodynamic perspective, the simulated interaction energy per CR molecule increased from approximately −126 kJ mol^−1^ at low coverage to −176 kJ mol^−1^ at saturation. These values were obtained from Grand Canonical Monte Carlo simulations and reflect the total non-covalent interaction energy per adsorbed molecule, encompassing electrostatic, hydrogen bonding, dispersion, and π–π stacking contributions. Although these energies exceed typical experimental isosteric heats of physisorption (commonly in the 20–80 kJ mol^−1^ range), they do not represent classical thermodynamic enthalpies. Instead, they represent simulated interaction energies that capture multi-contact adsorption configurations. Each CR molecule interacts simultaneously with multiple functional groups across a structurally and chemically heterogeneous surface, resulting in cumulative stabilization that naturally yields higher values than those derived from macroscopic experimental averaging. These findings collectively suggest that while the adsorption process is governed by strong physical interactions, it remains non-covalent and reversible, as further supported by FTIR analysis, regeneration experiments, and the absence of chemical transformation during adsorption.

This reversibility, combined with the high adsorption capacity and the synergistic combination of interactions, underscores the potential of OSP as a sustainable and cost-effective adsorbent for wastewater treatment applications. Furthermore, its high potential for regeneration and reuse make it an environmentally friendly solution for addressing dye pollution.

#### Proposed Adsorption Mechanism of Congo Red onto Orange Seed Powder

**Figure 9:**
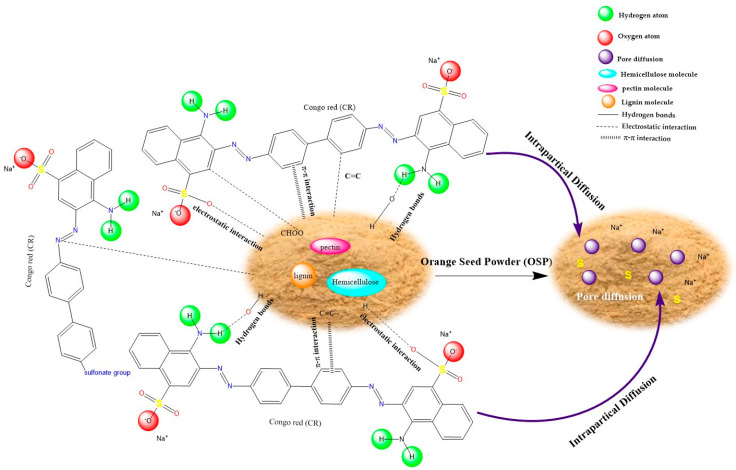
Schematic representation of the adsorption mechanism of Congo red onto orange seed powder.

Based on the complementary insights gained from FTIR, SEM-EDS analysis, adsorption isotherms, kinetic modeling, and Grand Canonical Monte Carlo (GCMC) simulations, a comprehensive adsorption mechanism for Congo red (CR) onto orange seed powder (OSP) is proposed and schematically illustrated in Figure 9. The adsorption process involves a synergistic combination of multiple molecular interactions and transport phenomena. Under optimal acidic conditions (pH 4), the protonated hydroxyl and carboxyl groups of OSP’s lignocellulosic components (pectin, hemicellulose, and lignin) establish strong electrostatic attractions with the anionic sulfonate groups (-SO_3_^−^) of CR. Concurrently, hydrogen bonding occurs between the oxygen-containing functional groups of OSP and the amino and sulfonate groups of the dye molecule. Additionally, π–π stacking interactions between the aromatic rings of CR and the phenolic moieties present in the lignin fraction of OSP further contribute to the adsorption affinity. From a kinetic perspective, the adsorption mechanism is governed by a two-step transport process: an initial rapid external mass transfer (film diffusion) of CR molecules to the biosorbent surface, followed by intraparticle (pore) diffusion of the dye into the porous lignocellulosic matrix until equilibrium is attained. This multidimensional representation, integrating the heterogeneous surface chemistry of OSP with the synergistic molecular interactions and diffusional phenomena, is fully consistent with the experimental characterization results and provides a molecular-level understanding of the excellent adsorption performance of this sustainable biosorbent.

### 2.7. Regeneration and Reusability Performance of Adsorbent

Reusability and regeneration of sorbents are crucial for scaling up adsorption processes and ensuring their practical application in industrial settings. Regeneration agents should be cost-effective, environmentally safe, and capable of maintaining the structural integrity of the biosorbent while enabling multiple reuse cycles [88]. The choice of desorption agent depends on both the physicochemical properties of the biosorbent and the adsorption mechanism of the target pollutant [89].

**Figure 10:**
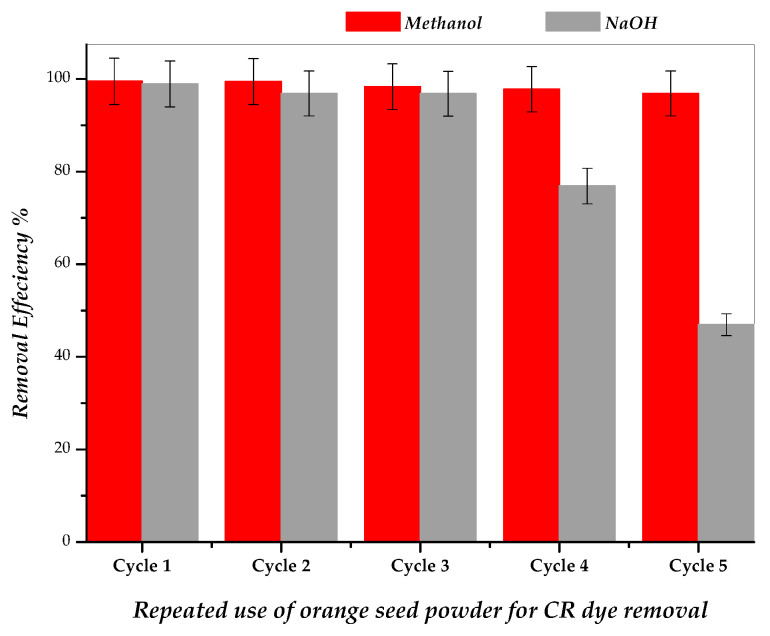
Regeneration and reuse of orange seed powder (OSP) over five consecutive adsorption–desorption cycles using two regeneration protocols (methanol and 0.1 N NaOH) under optimal conditions (C_0_ = 400 mg L^−1^, t = 120 min, pH = 4, adsorbent dosage = 3 g L^−1^, T = 25 °C and particle size = 125–500 µm).

In this study, methanol and 0.1 N NaOH were used as desorption agents for Congo red (CR) removal (Figure 10). The results clearly indicate that methanol is more effective than NaOH for regenerating OSP. The adsorption removal efficiency of OSP regenerated with methanol remained above 96.85% even after five consecutive cycles, decreasing only slightly from 99.47% in the first cycle to 96.85% in the fifth cycle. In contrast, OSP regenerated with 0.1 N NaOH showed a significant decline in performance, dropping from 98.89% in the first cycle to 46.95% in the fifth cycle. These findings demonstrate that methanol efficiently disrupts physical interactions such as hydrogen bonding and van der Waals forces between CR molecules and the OSP surface, allowing effective regeneration over multiple cycles.

Generally, regeneration of biosorbents is performed at low temperatures to reduce operational costs and maintain structural integrity. Both organic and inorganic regeneration agents can achieve high regeneration performance; however, changes in the pore structure and availability of active sites may influence the effectiveness of chemical regeneration. Biosorbents loaded with organic pollutants such as dyes are relatively easy to regenerate due to the predominance of physical interactions. As expected, regeneration efficiency generally decreases with increasing cycle number, although optimization of operational parameters can mitigate this decline. Although solvents such as methanol, ethanol, and acetone are effective for regeneration, the use of environmentally friendly and inexpensive alternatives should be explored for large-scale applications due to potential toxicity and economic considerations [88,90,91].

## 3. Materials and Methods

### 3.1. Materials

Sweet orange seeds were obtained from damaged *Citrus sinensis* fruits discarded by consumers and from industrial processing chains in Constantine, Algeria. Congo red dye (C_32_H_22_N_4_O_6_S_2_Na_2_, MW = 696.7 g·mol^−1^, λ_max_ = 500 nm) was purchased from Merck (Germany). Hydrochloric acid (HCl) and sodium hydroxide (NaOH), both of analytical grade, were supplied by Sigma-Aldrich. All solutions were prepared using distilled water.

### 3.2. Preparation of Orange Seed Powder (OSP) Adsorbent

The sweet orange seeds described above were thoroughly washed with tap water to remove surface impurities and soluble contaminants. The seeds were then separated from the fruit pulp using a fruit-pressing system equipped with a dedicated filter. The recovered seeds were subsequently used for the preparation of the biosorbent material.

For adsorbent preparation, the seeds were air-dried at room temperature for one week, ground, and sieved to obtain a particle size range of 125–500 µm, ensuring uniformity and controlled moisture content. The resulting powder was rinsed with distilled water to remove residual impurities, re-dried under the same conditions, and stored in airtight polyethylene bottles to preserve stability until further use.

### 3.3. Structural and Morphological Characterizations of the Adsorbent

The OSP sample used for post-adsorption characterization (FTIR, SEM-EDS) was obtained from a batch experiment conducted under optimal conditions: initial CR concentration of 400 mg L^−1^, OSP dosage of 3 g L^−1^, pH of 4, contact time of 120 min, and temperature of 25 °C.

#### 3.3.1. Fourier Transform-Infrared Spectroscopy (FTIR)

Fourier transform infrared spectroscopy was employed to characterize the surface functional groups of the adsorbent. Briefly, 2 mg of orange seed powder (OSP) was mixed and finely ground with 98 mg of spectroscopic grade KBr. The mixture was then pressed into a pellet (1 cm diameter × 2 mm thickness). FTIR spectra were recorded before and after CR adsorption, using the spectrometer (FTIR-2000, PerkinElmer) operating in the wavenumber range of 4000–500 cm^−1^ at a resolution of 4 cm^−1^.

#### 3.3.2. Scanning Electron Microscopy (SEM) and Energy Dispersive X-Rays Spectroscopy (EDS)

Scanning electron microscopy (SEM) was used to investigate the surface morphology of OSP both before and after CR adsorption. The analysis was performed using a field-emission scanning electron microscope (FE-MEB, Quattro S, Thermo Fisher Scientific) equipped with an energy dispersive X-rays spectroscopy (EDS) detector (EDAX). The samples were mounted on aluminum stubs and examined at a magnification of 500×, with a spatial resolution of 50 µm.

### 3.4. Equilibrium Adsorption Experiments

Adsorption experiments were investigated using a batch mode to evaluate the influence of operational parameters on CR adsorption, including initial CR concentration (100, 200, 300, and 400 mg L^−1^), adsorbent dosage (1, 2, 3, 4, and 5 g L^−1^), initial pH (2, 4, 5, 7, 9, and 11), and contact time. A contact time of 120 min was chosen for all batch equilibrium experiments to ensure that a stable equilibrium was reached under all conditions, even though the initial rapid uptake was largely completed within the first 20 min for most experimental conditions.

In each adsorption experiment, 200 mL of CR solution at the desired concentration and pH was mixed with a known amount of adsorbent in a 250 mL conical flask. The mixture was magnetically stirred for 2 h at a constant stirring speed of 250 rpm. The initial pH was 6.95 (without any adjustment), and then adjusted using hydrochloric acid (0.1 M HCl) and sodium hydroxide (0.1 M NaOH). All experiments were performed at a controlled temperature of 25 ± 1 °C. Samples were collected at predetermined intervals and filtered through 0.45 μm filter paper to retain the OSP, and the absorbance was measured at λ_max_ = 500 nm.

The adsorption capacity of OSP $Q_{t}$ (mg of CR/g of OSP) and the removal efficiency, *R* (%), at time *t* were determined from the absorbance using the following Equations (6) and (7), respectively:

(6) $$Q_{t}(mg/g)=\frac{\left(C_{i}-C_{t}\right)}{m}\times V$$

(7) $$R(\%)=\frac{\left(C_{i}-C_{t}\right)}{C_{i}}\times 100$$

where *C_i_* and *C_t_* are the initial and final CR concentrations (mg L^−1^) at time *t*, *m* is the adsorbent mass (g), and *V* is the solution volume (L).

To comprehensively analyze the adsorption behavior, multiple kinetic and equilibrium models were employed, including the pseudo-first-order and pseudo-second-order models, the Langmuir and Freundlich isotherms, the Elovich model, and the intraparticle diffusion model. These models provided insights into the rate-limiting steps and the overall mechanism governing the adsorption of CR onto OSP.

### 3.5. Statistical Analysis

Adsorption experiments were performed in triplicate to ensure the reliability and reproducibility of the results. The reported values are expressed as mean ± standard deviation (*n* = 3). All batch adsorption data represent the average of three independent experiments.

The model parameters were determined using linear regression analysis of the experimental data. The goodness of fit was evaluated using the correlation coefficient (*R*^2^) and the error function (Chi-square, χ^2^), calculated according to Equation (8). Lower χ^2^ values and an *R*^2^ values closer to 1 indicate a better fit of the model to the experimental data.

(8) $$\chi^{2}=\sum_{i=1}^{n}\frac{{\left(Q_{e,exp}-Q_{e,cal}\right)}^{2}}{Q_{e,cal}}$$

where *n* is the number of replicate experiments, and $Q_{e,exp}$ and $Q_{e,cal}$ are the experimental and calculated adsorption capacities at equilibrium (mg/g), respectively.

### 3.6. Molecular Simulations

#### 3.6.1. Modeling OSP, Water, CR and Force Fields

An amorphous model of orange seed powder (OSP) was constructed, comprising albumin protein (10%), cellulose (20%), lignin (10%), amylose (20%), glutelin protein (10%), and amylopectin (30%) (Figure S1, Supplementary Information). This composition reflects the typical biochemical constituents of orange seeds reported in the literature, where polysaccharides provide structural integrity and energy storage, cellulose and lignin contribute to mechanical rigidity, and proteins support metabolic and storage functions [39,40,92,93].

The amorphous structure was generated using Monte Carlo simulations to minimize steric clashes and replicate realistic molecular packing. Molecules were randomly placed within a periodic simulation box, followed by energy minimization using the conjugate gradient method and packing optimization via the Metropolis algorithm [94,95].

Partial charges of Congo red (CR) were computed using the Electrostatic Potential (ESP) method in DMol^3^ (GGA/PW91 functional, DNP basis set) to accurately model electrostatic interactions [96]. Co-adsorption of water and CR was simulated using the TIP4P/2005 model for water, and flexible DREIDING force field parameters for the biomolecular matrix [97,98]. Non-bonded interactions were described using Lennard-Jones (12-6) and Coulombic potentials, with cross-interactions calculated via the Berthelot mixing rule using the following Equation (9) [99,100].

(9) $$V_{\left(r_{ij}\right)}=\frac{q_{i}q_{j}}{4\pi \varepsilon_{o}r_{ij}}+4\varepsilon \left[{\left(\frac{\sigma }{r_{ij}}\right)}^{12}-{\left(\frac{\sigma }{r_{ij}}\right)}^{6}\right]$$

where *ε* is the depth of the potential well, *σ* is the distance at which the interatomic potential is zero, and $r_{ij}$ is the distance separating the atoms [100]. This rigorous modeling framework enhances the understanding of adsorption mechanisms in complex biological materials by providing critical insights into how molecular composition and structural configuration govern adsorption efficiency and capacity.

Force field parameters for bonded interactions (bonds, angles, and torsions) as well as non-bonded interactions are summarized in Tables S1 and S2 (Supplementary Information). The OSP framework was treated as fully flexible in order to capture structural responses induced by adsorption. This modeling strategy ensures a realistic representation of molecular interactions and yields reliable mechanistic insights into the adsorption efficiency and capacity of OSP.

#### 3.6.2. Grand Canonical Monte Carlo Approach

To gain insight into the interaction strength between CR molecules and the OSP matrix, the average potential energy change per adsorbate molecule was calculated from the GCMC trajectories. This value, hereafter referred to as the simulated interaction energy, is defined as the difference between the average potential energy of an adsorbate molecule in the adsorbed state and its energy in an ideal gas reference state. It reflects the combined contributions of all non-bonded interactions (Coulombic and Lennard-Jones) described by the force field. It should be noted that these simulated energies represent the total binding strength under the specific model conditions and are not directly equivalent to experimentally determined isosteric enthalpies of adsorption, which typically reflect an average over all adsorption sites and experimental conditions.

For the co-adsorption studies, water molecules (modeled using TIP4P/2005) and CR molecules were introduced into an equilibrated amorphous OSP matrix. To replicate realistic adsorption conditions, Grand Canonical Monte Carlo (GCMC) simulations were employed to model particle exchange with a reservoir at constant chemical potential. All simulations were performed using the Dl_Monte software package, with the simulation box defined as a single periodic unit cell. Each simulation consisted of 1 × 10^6^ Monte Carlo steps for both the equilibration and production stages.

Electrostatic interactions were calculated using the Ewald summation technique [101] to ensure accurate treatment of long-range forces, while Lennard-Jones interactions were computed using a cut-off distance of 12 Å to account for short-range interactions. This simulation framework proves highly efficient for adsorption isotherm analysis since it permits direct determination of uptake capacities at different chemical potentials. Consequently, it facilitates meaningful comparison with experimental measurements, thereby validating the simulation results and providing a deeper understanding of the adsorption behavior.

GCMC simulations were conducted under both high-loading conditions, corresponding to experimental saturation levels, and low-loading conditions to explore the fundamental adsorption mechanisms at low surface coverage. This dual-condition strategy provides valuable insights into both the overall adsorption capacity and the molecular interactions governing the adsorption process.

### 3.7. Regeneration and Reusability Procedureof the Adsorbent

The reusability of orange seed powder (OSP) for Congo red (CR) adsorption was evaluated over five consecutive adsorption–desorption cycles using two regeneration protocols: (i) treatment with methanol, and (ii) treatment with 0.1 N NaOH as desorption agents [88]. In the first protocol, dye-loaded OSP was treated with methanol (50 mL) at 25 °C for 60 min under magnetic stirring at 150 rpm to disrupt hydrogen bonding and van der Waals interactions between CR molecules and the adsorbent surface. In the second protocol, OSP was treated with 0.1 N NaOH under identical conditions. After each desorption step, OSP was rinsed, dried at 60 °C for 12 h, and reused in the subsequent adsorption cycle. The regeneration performance was evaluated by comparing the CR removal efficiency after each cycle. Regeneration efficiency RE (%) was calculated using Equation (10):

(10) $$RE\;(\%)=\frac{Q_{e,n}}{Q_{e,1}}\times 100$$

where $Q_{e,1}$ and $Q_{e,n}$ (mg g^−1^) represent the adsorption capacities of fresh and regenerated OSP after the *n*th cycle, respectively.

## 4. Conclusions

The aim of this work was to valorize a natural agro-food lignocellulosic residue, namely orange seeds, as a low-cost biosorbent prepared without any thermal or chemical treatment for the removal of Congo red (CR) from aqueous solutions. The adsorption mechanism and adsorptive performance were thoroughly investigated through both experimental and theoretical approaches. The experimental results were well represented by both the Langmuir and Freundlich isotherms, suggesting that monolayer and multilayer adsorption processes occur simultaneously at 25 °C.

Kinetic analysis revealed that the pseudo-second-order model best fitted the experimental data, as evidenced by the close agreement between experimental and calculated adsorption capacities, suggesting that physical interactions (hydrogen bonding, van der Waals) are dominant, while chemisorption may contribute. Furthermore, the Elovich and intra-particle diffusion models indicated that the adsorption process is governed by a combination of surface adsorption and diffusion mechanisms. Grand Canonical Monte Carlo (GCMC) simulations were conducted to complement the experimental findings, providing molecular-level insight into the adsorption mechanism and further confirming the effectiveness of orange seed powder as a sustainable and cost-effective adsorbent for wastewater treatment.

Orange seed powder (OSP) also demonstrated excellent regeneration and reusability over five consecutive adsorption–desorption cycles, retaining more than 96% of its initial Congo red removal efficiency when regenerated with methanol. These results highlight the strong potential of OSP as a stable, reusable and sustainable biosorbent suitable for practical wastewater treatment applications.

Overall, this study successfully demonstrates the valorization of orange seed waste into an efficient, low-cost biosorbent for Congo red removal. The simple preparation process, involving only drying and grinding, enhances the added value of this agricultural by-product and offers a sustainable and environmentally friendly alternative to conventional engineered adsorbents. The large-scale application of unmodified OSP, either alone or integrated with existing wastewater treatment technologies, appears highly promising due to its efficiency, sustainability, cost-effectiveness, and minimal environmental impact.

While laboratory results demonstrate that Orange Seed Powder is a promising biosorbent, further investigations are required to facilitate its industrial implementation. OSP presents notable advantages, including low-cost preparation from abundant citrus waste and potential scalability. However, aspects such as replacing methanol with more environmentally friendly regeneration agents, evaluation under continuous-flow column systems, and validation using real industrial wastewater remain to be explored. In addition, a comprehensive lifecycle assessment of spent OSP is recommended to ensure long-term environmental sustainability. Addressing these challenges will help bridge the gap between laboratory research and practical application.

## Figures and Tables

**Table 1:** Langmuir and Freundlich isotherm parameters for the adsorption of CR onto OSP. (Experimental conditions: OSP/CR: 3 g/L, pH = 4, T = 25 °C, stirring speed = 250 rpm, particle size = 125–500 µm).

Isotherm Model	Parameters	Initial Concentration (mg L^−1^)			
		100	200	300	400
Langmuir model	$Q_{e,exp}(mg.g^{-1})$	32.7062	65.0424	97.5106	130.1330
	$Q_{e,cal}(mg.g^{-1})$	35.6976	75.8059	100.4839	116.2684
	$b(L.{mg}^{-1})$	0.0852			
	$Q_{m}(mg.g^{-1})$	258.3979			
	$R_{L}$	0.1050	0.0554	0.0376	0.0285
	$R^{2}$	0.99981			
	$\chi^{2}$	0.2506	1.5282	0.0879	1.6533
Freundlich model	$Q_{e,exp}(mg.g^{-1})$	32.7062	65.0424	97.5106	130.1330
	$Q_{e,cal}(mg.g^{-1})$	30.2384	69.3875	100.7262	125.4191
	n	1.1457			
	$\frac{1}{n}$	0.8727			
	Kf (mg.g−1)(L.mg−1)1n	17.4188			
	$R^{2}$	0.99925			
	$\chi^{2}$	0.2014	0.2720	0.1026	0.1771

Footnotes: $Q_{m}$: Maximum adsorption capacity (monolayer); *b*: Langmuir affinity constant; $R_{L}$: Separation factor (dimensionless); n: Adsorption intensity; $K_{f\;}$: Freundlich capacity constant.

**Table 2:** Comparison of Langmuir separation factor ($R_{L})$, Freundlich constant (n), and maximum adsorption capacity ${(Q}_{m}$) for Congo red adsorption onto various adsorbents reported in the literature.

Adsorbent	$C_{0}$ $\left(mg\;L^{-1}\right)$	$R_{L}$(Langmuir)	*n* (Freundlich)	Isotherm Model	$Q_{m}$ $\left(mg\;g^{-1}\right)$	Ref.
Jujuba seed	25–100	0.0124	1.6639	Langmuir	55.56	[24]
Eucalyptus (*Eucalyptus globulus*) sawdust	10–30	0.605–0.864	2.53	Redlich and Peterson (R–P)	31.25	[25]
Modified Orange peel Powder	50–300	0.26	2.89	Langmuir	107	[26]
Defatted orange seed powder	Real wastewater	—	—	—	~95% removal	[32]
Fe_2_O_3_/activated charcoal (de-oiled orange seed)	20–100	0.081	3.968	Freundlich	9.21	[33]
Coir pith	20–80	0.06–0.21	3.39–4.78	Langmuir and Freundlich	6.72	[43]
Phoenix dactylifera seeds	20–120	0.51	1.858	Langmuir	61.72	[45]
Cabbage Waste Powder	4.88–48.76	0.0801–0.0086	1.568	Langmuir	2.313	[46]
Ackee apple (*Blighia sapida*) seeds	50–300	0.43	2.16	Langmuir	161.89	[53]
Chir pine (*Pinus roxburghii*) sawdust	20–100	0.113	2.320	Freundlich	5.310	[62]
Rubber seeds (*Hevea brasiliensis*)	20–100	—	3.93	Langmuir	9.82	[63]
Orange seeds powder	100–400	0.0285–0.105	1.1457	Langmuir and Freundlich	258.39	This study

Note: ‘—’ indicates ‘not mentioned’.

**Table 3:** Kinetic parameters obtained from pseudo-first order, pseudo-second order, Elovich and Weber–Morris (intra-particle diffusion) models. (Experimental conditions: OSP/CR: 3 g/L, pH = 4, T = 25 °C, stirring speed = 250 rpm, particle size = 125–500 µm.)

	Kinetic Parameters	Concentration (mg L^−1^)			
		100	200	300	400
Pseudo-first-order kinetic (Lagergren model)	$Q_{e,exp}(mg\;g^{-1})$	32.706	65.042	97.560	130.911
	$Q_{e,cal}(mg\;g^{-1})$	2.267	4.023	3.637	2.390
	$K_{1}({min}^{-1})$ (-)	0.0002	0.0004	0.0003	0.0004
	*R*^2^ (-)	0.872	0.826	0.860	0.891
	$\chi^{2}$	408.59	925.34	2425.43	6908.33
Pseudo-second-order kinetic (Ho model)	$Q_{e,cal}(mg\;g^{-1})$	33.277	65.231	97.560	131.061
	$K_{2}(g\;{mg}^{-1}\;{min}^{-1})$	0.0264	0.0123	0.0098	0.0711
	$R^{2}$	0.9999	0.9997	0.9998	1
	$\chi^{2}$	0.0098	0.0005	0.0000	0.0001
Elovich	$\alpha (mg\;g^{-1}\;{min}^{-1})$	2.157$\;\times \;10^{13}$	1.952$\;\times \;10^{17}$	2.797$\;\times \;10^{19}$	8.136$\;\times \;10^{88}$
	$\beta (g\;{mg}^{-1})$	1.075	0.686	0.505	1.601
	$R^{2}$	0.916	0.884	0.928	0.917
Intra-particle diffusion model (Weber–Morris)	$k_{d}(mg\;g^{-1}\;{min}^{-0.5})$(-)	0.289	0.502	0.628	0.175
	$C_{d}(mg\;g^{-1})$	30.062	59.475	90.253	129.218
	$R^{2}$	0.872	0.902	0.928	0.708

Footnotes: $K_{1}({min}^{-1})$: Lagergren kinetic constant. $K_{2}(g\;{mg}^{-1}\;{min}^{-1})$: rate constant of pseudo-second-order model. $\chi^{2}$: Chi-square (statistical factor). $\alpha (mg\;g^{-1}\;{min}^{-1})$: constant about the initial adsorption rate. $\beta (g\;{mg}^{-1})$: constant refers to the extent of surface coverage, and activation energy for chemisorption. $C_{d}(mg\;g^{-1})$: Morris–Weber constant, proportional to the boundary layer thickness). $k_{d}(mg\;g^{-1}\;{min}^{-0.5})$: intraparticle diffusion rate constant.

## Data Availability

The original contributions presented in this study are included in the article/Supplementary Material. Further inquiries can be directed to the corresponding author.

## Supplementary Materials

The following supporting information can be downloaded at: https://www.mdpi.com/article/10.3390/molecules31071152/s1. Figure S1: Schematic representation of the individual structural components integrated to form the amorphous matrix of the orange seed.; Table S1: Detailed force field parameters for each atom type within the molecules comprising the amorphous structure, including bond stretching, angle bending, torsional potentials, and non-bonded Lennard-Jones parameters (ε and σ). These values, derived from the UFF and Dreiding force fields, are essential for accurately modeling the molecular interactions within the biomolecules.; Table S2. Comprehensive Lennard-Jones (LJ) parameters for each atom type within the amorphous structure, detailing the co-adsorption behaviour of water (modelled using TIP4P/2005) and Congo Red. Derived from the Dreiding Force Field, these parameters accurately capture the non-bonded interactions essential for analysing adsorption behaviour in the biomolecular matrix.
