# Supplementary material for: Orange Seed Powder as a Novel Biosorbent for Congo Red Removal: Adsorption Mechanism, Isotherms, Kinetics, and Molecular Simulations
Source: Molecules. 2026 Mar 31;31(7):1152. doi: 10.3390/molecules31071152 (PMC13074945; doi:10.3390/molecules31071152)
Supplement: Supplementary file 1 [file molecules-31-01152-s001.zip › molecules-4157889-supplementary.pdf]

Supplementary Information (SI)

Manuscript ID: molecules-4157889

Title: Orange Seed Powder as a Novel Biosorbent for Congo Red Removal: Adsorption Mechanism, Isotherms, Kinetics, and Molecular Simulations

1.1. Construction of a comprehensive computational matrix for the orange seed model

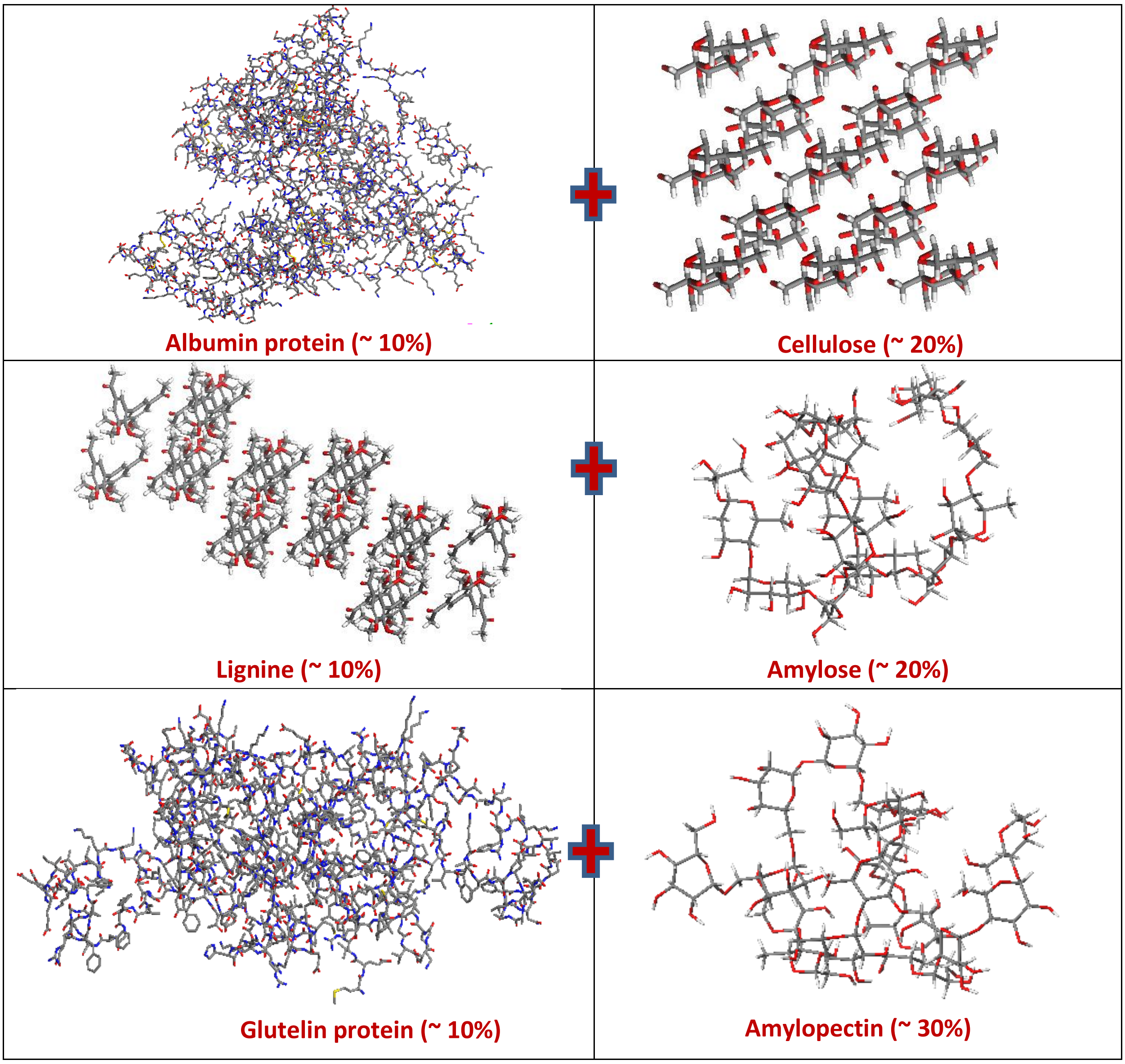

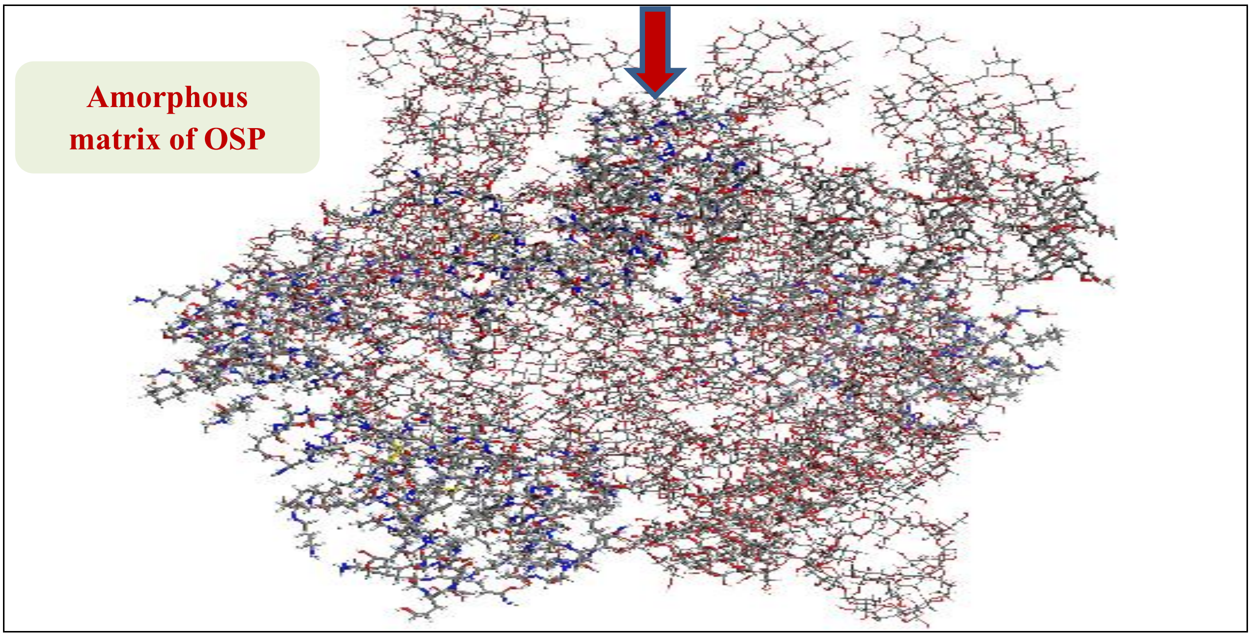

Figure S1. Schematic representation of the individual structural components integrated to form the amorphous matrix of the orange seed.

1.2. Force fields parameters

Table S1. Detailed force field parameters for each atom type within the molecules comprising the amorphous structure, including bond stretching, angle bending, torsional potentials, and non-bonded Lennard-Jones parameters (ε and σ). These values, derived from the UFF and Dreiding force fields, are essential for accurately modeling the molecular interactions within the biomolecules.

Molecule	Atom Type	Bond Stretching (kcal/mol/Å²)	Angle Bending (kcal/mol/rad²)	Torsional Potential (kcal/mol)	Non-bonded LJ ε (kcal/mol)	Non-bonded LJ σ (Å)
Albumin	C	350	50	1.75	0.10	3.5
	H	340	45	1.6	0.05	2.5
	O	360	55	1.9	0.12	3.2
	N	370	60	2.1	0.15	3.4
	S	375	58	2.2	0.18	3.6
Cellulose	C	320	45	1.75	0.12	3.4
	H	310	40	1.6	0.06	2.4
	O	330	50	1.9	0.14	3.1
Lignin	C	300	40	1.5	0.15	3.3
	H	290	35	1.35	0.07	2.3
	O	310	45	1.65	0.17	3.0
	S	320	48	1.75	0.19	3.2
Amylose	C	310	42	1.5	0.13	3.4
	H	300	38	1.35	0.06	2.4
	O	320	46	1.15	0.15	3.3
Glutelin	C	340	48	1.85	0.10	3.5
	H	330	44	1.7	0.05	2.5
	O	350	52	2.0	0.12	3.2
	N	360	54	2.15	0.14	3.3
Amylopectin	C	315	43	1.65	0.14	3.4
	H	305	39	1.5	0.07	2.4
	O	325	47	1.75	0.16	3.1

Table S2. Comprehensive Lennard-Jones (LJ) parameters for each atom type within the amorphous structure, detailing the co-adsorption behaviour of water (modelled using TIP4P/2005) and Congo Red. Derived from the Dreiding Force Field, these parameters accurately capture the non-bonded interactions essential for analysing adsorption behaviour in the biomolecular matrix.

Molecule	Atom Type	LJ ε with Water (kcal/mol)	LJ σ with Water (Å)	LJ ε with Congo Red (kcal/mol)	LJ σ with Congo Red (Å)
Albumin	C	0.12	3.55	0.15	3.60
	H	0.06	2.75	0.07	2.80
	O	0.14	3.25	0.17	3.30
	N	0.17	3.35	0.20	3.40
	S	0.20	3.50	0.22	3.55
Cellulose	C	0.14	3.45	0.17	3.50
	H	0.07	2.65	0.08	2.70
	O	0.16	3.15	0.19	3.20
Lignin	C	0.17	3.40	0.20	3.45
	H	0.08	2.55	0.09	2.60
	O	0.19	3.05	0.21	3.10
	S	0.21	3.20	0.24	3.25
Amylose	C	0.15	3.45	0.18	3.50
	H	0.07	2.65	0.08	2.70
	O	0.18	3.20	0.20	3.25
Glutelin	C	0.12	3.50	0.15	3.55
	H	0.06	2.70	0.07	2.75
	O	0.14	3.25	0.17	3.30
	N	0.16	3.30	0.19	3.35
Amylopectin	C	0.16	3.45	0.19	3.50
	H	0.08	2.65	0.09	2.70
	O	0.18	3.15	0.21	3.20
